# Author Correction: NMNAT2 is a druggable target to drive neuronal NAD production

**DOI:** 10.1038/s41467-024-52439-7

**Published:** 2024-09-17

**Authors:** James R. Tribble, Melissa Jöe, Carmine Varricchio, Amin Otmani, Alessio Canovai, Baninia Habchi, Evangelia Daskalakis, Romanas Chaleckis, Andrea Loreto, Jonathan Gilley, Craig E. Wheelock, Gauti Jóhannesson, Raymond C. B. Wong, Michael P. Coleman, Andrea Brancale, Pete A. Williams

**Affiliations:** 1 Department of Clinical Neuroscience, Division of Eye and Vision, St. Erik Eye Hospital; Karolinska Institutet, Stockholm, Sweden; 2 School of Pharmacy and Pharmaceutical Sciences; Cardiff University, Cardiff, Wales UK; 3 Department of Biology, University of Pisa, 56127 Pisa, Italy; 4 Unit of Integrative Metabolomics, Institute of Environmental Medicine, Karolinska Institute, Stockholm, Sweden; 5 Department of Respiratory Medicine and Allergy, Karolinska University Hospital, Stockholm, Sweden; 6 C2VN, INRAE, INSERM, Aix Marseille University, 13007 Marseille, France; 7 Gunma Initiative for Advanced Research (GIAR), Gunma University, Maebashi, Japan; 8 John van Geest Centre for Brain Repair, Department of Clinical Neurosciences; University of Cambridge, Cambridge, UK; 9 School of Medical Sciences and Save Sight Institute, Charles Perkins Centre, Faculty of Medicine and Health, The University of Sydney, Sydney, NSW Australia; 10 Department of Clinical Sciences, Ophthalmology, Umeå University, 901 85 Umeå, Sweden; 11 Wallenberg Centre of Molecular Medicine, Umeå University, 901 85 Umeå, Sweden; 12 Centre for Eye Research Australia, Royal Victorian Eye and Ear Hospital, East Melbourne, Australia; 13 Ophthalmology, Department of Surgery, University of Melbourne, East Melbourne, VIC Australia; 14 Vysoká škola chemicko-technologická v Praze, Prague, Czech Republic

**Keywords:** Diseases of the nervous system, Retina, Drug development

Correction to: *Nature Communications* 10.1038/s41467-024-50354-5, published online 24 July 2024

In this article Figure 1 was an older version, which does not match the figure legend and duplicates panels from Figure 2. The updated and correct Figure 1 is below.
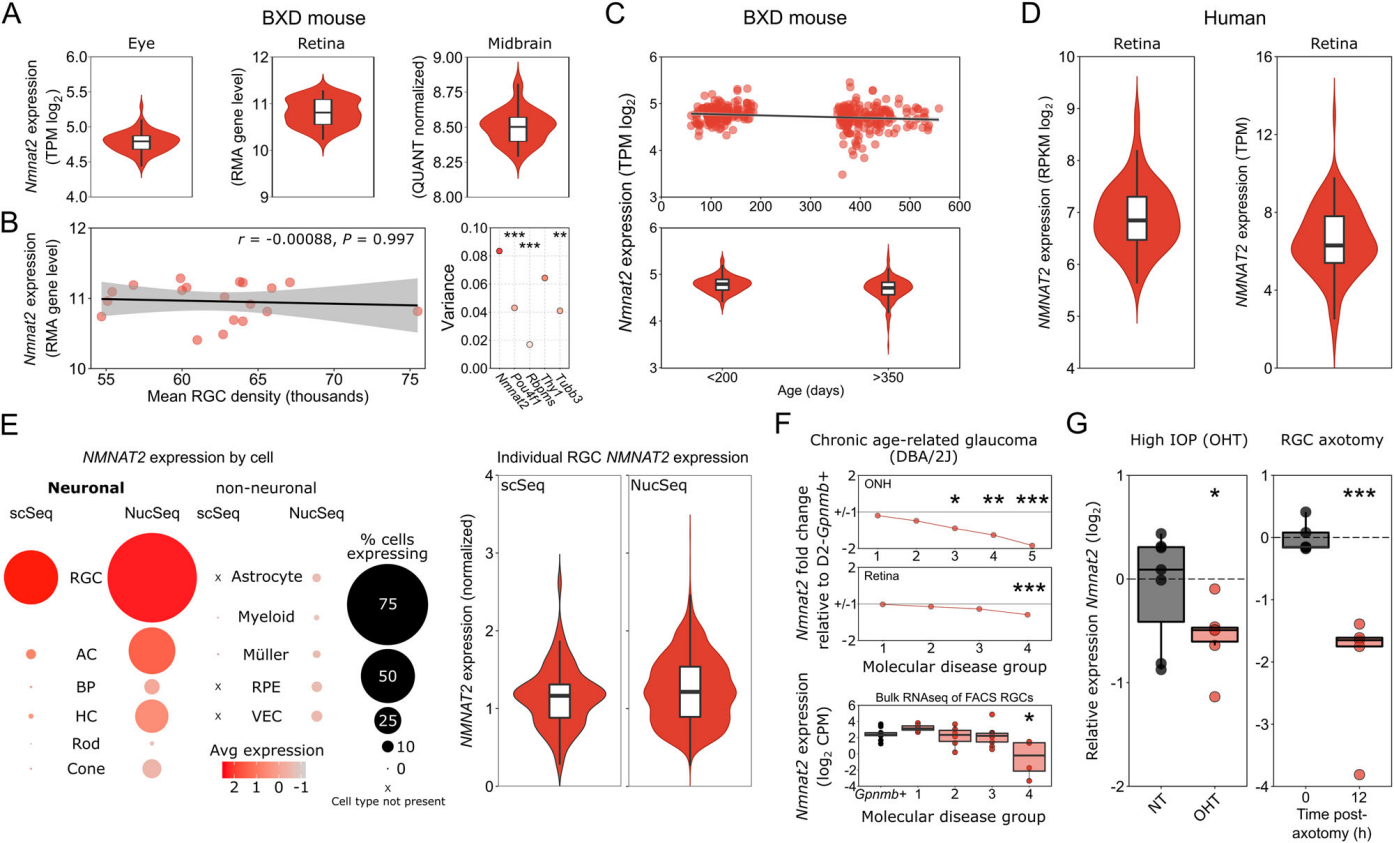


Data has not changed, and no text was amended. The original article has been corrected.

