# Supplementary material for: NMNAT2 is a druggable target to drive neuronal NAD production
Source: Nat Commun. 2024 Jul 24;15:6256. doi: 10.1038/s41467-024-50354-5 (PMC11269627; doi:10.1038/s41467-024-50354-5)
Supplement: Supplementary file 1 — Supplementary Information [file 41467_2024_50354_MOESM1_ESM.pdf]

Title: NMNAT2 is a druggable target to drive neuronal NAD production

Supplementary Information

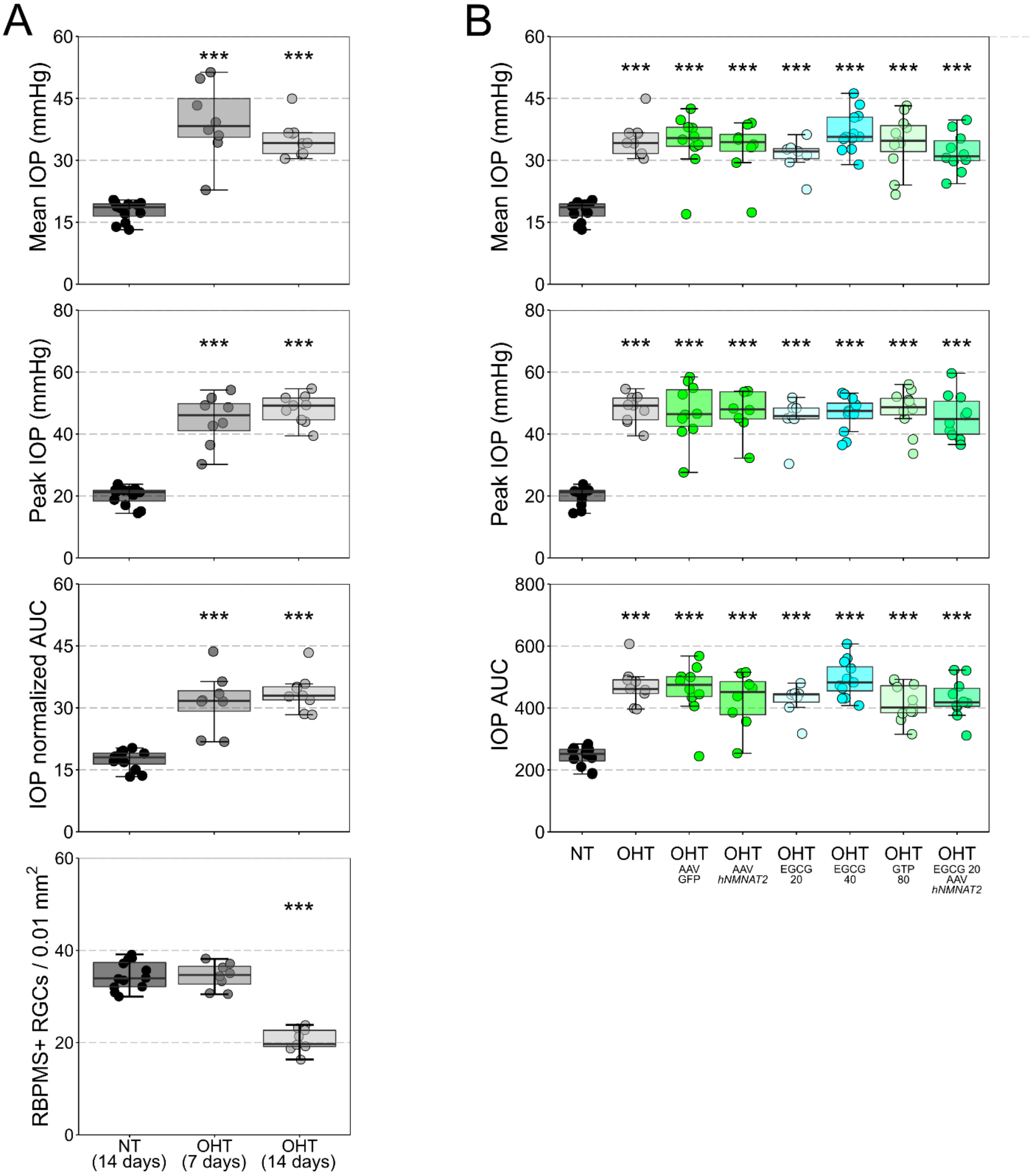

Fig. S1. Rat IOP profiles and RGC survival at 7 days post OHT induction

(**A**) To determine whether *Nmnat2* expression is reduced in the rat in response to ocular hypertension (OHT) but in the absence of RGC death we determined the degree of RGC loss following 7 days of OHT (n = 8 eyes). At this time-point IOP is high, with a mean, peak, and cumulative IOP (AUC; normalized here to account for duration) which is not statistically different to 14 days of OHT (n = 9 eyes). However, at this 7 day time-point there is no significantly detectable change in RGC density compared to normotensive (NT) controls (n = 12 eyes). By 14 days, a significant loss of RGC density has occurred compared to both 7 day OHT animals and NT controls. **B)** For all experimental rat cohorts mean, peak, and IOP AUC are significantly increased compared to NT controls. Mean, peak, and IOP AUC are not significantly different between any experimental cohorts that are OHT, supporting that IOP does not significantly influence any differences in RGC survival between groups. For OHT AAV GFP, n = 10 eyes; OHT *hNMNAT2*, n = 8 eyes; OHT EGCG 20 mg/kg/d, n = 7 eyes; OHT EGCG 40 mg/kg/d, n = 12 eyes; OHT GTP 80 mg/kg/d, n = 10 eyes; OHT *hNMNAT2* + EGCG 20 mg/kg/d, n = 11 eyes. * = $P < 0.05$, ** = $P < 0.01$, ***$P < 0.001$, NS = non-significant ($P > 0.05$); One-way ANOVA with *Tukey's* HSD. For box plots, the center hinge represents the median with upper and lower hinges representing the first and third quartiles; whiskers represent 1.5 times the interquartile range.

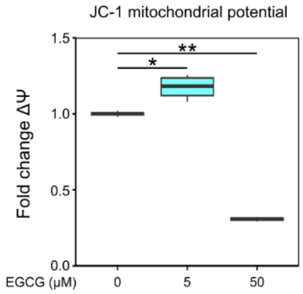

Fig. S2. EGCG effects on mitochondrial membrane potential

(A) The effects of EGCG on mitochondrial membrane potential were investigated. Dissociated cortical neurons were incubated with EGCG for 2 hours and JC-1 staining fluorescence was measured. At 5 μM EGCG significantly increased membrane potential relative to untreated controls, and at 50 μM EGCG significantly decreased membrane potential relative to untreated controls ($n = 4$ samples per condition). * = $P < 0.05$, ** = $P < 0.01$; One-way ANOVA with *Tukey's* HSD. For box plots, the center hinge represents the median with upper and lower hinges representing the first and third quartiles; whiskers represent 1.5 times the interquartile range.

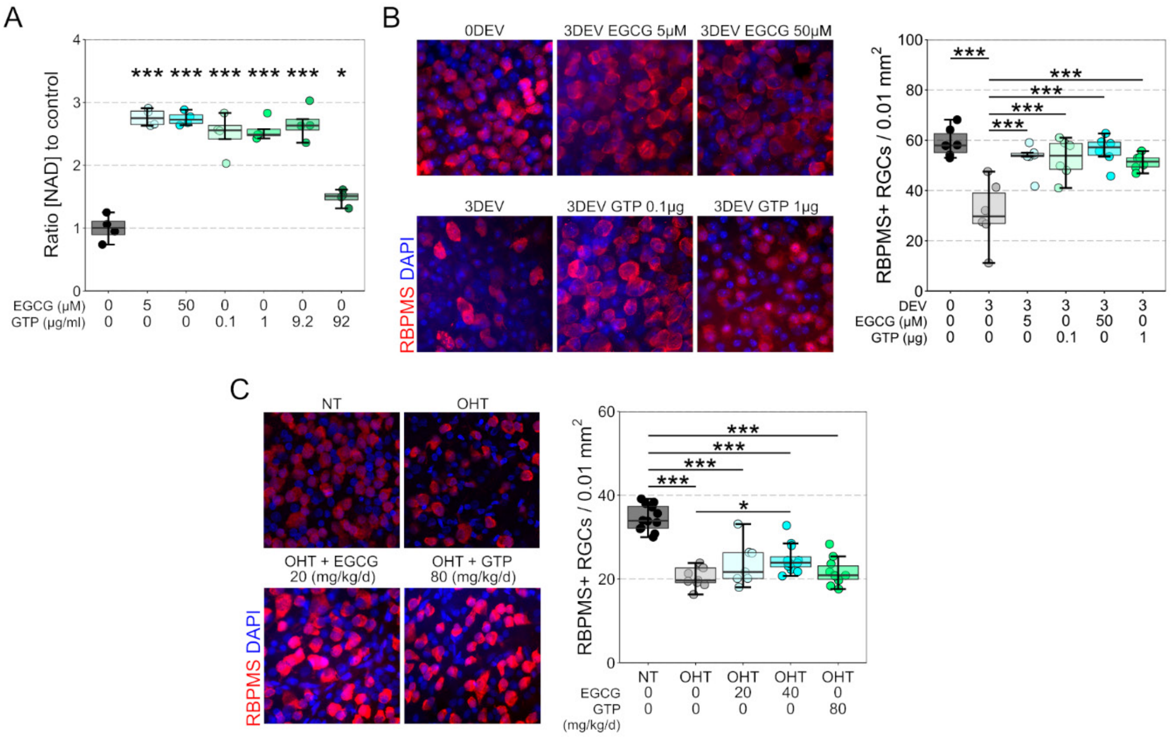

Fig. S3. EGCG compared to whole green tea polyphenols.

EGCG is a major green tea polyphenol and constitutes ~25-30% by weight of green tea polyphenol extract. A) Green tea polyphenol extract (GTP) significantly increases NAD in dissociated cortical neurons at concentrations where the EGCG content is comparable to pure EGCG (0.1 µg/ml and 1 µg/ml contains an equivalent EGCG content by weight to 5 and 50 µM EGCG). This effect is maintained up to 9.2 µg/ml but is significantly reduced at 92 µg/ml due to solution opacity interfering with luminescence signal. B) At 0.1 µg/ml and 1 µg/ml GTP provides neuroprotection against RGC loss following axotomy which is comparable to 5 and 50 µM EGCG. C) In a rat model of OHT, GTP at 80 mg/kg/d (equivalent to 20/mg/kg/d EGCG) did not provide significant neuroprotection over untreated OHT controls. Testing of higher GTP doses was not conducted since at 80 mg/kg/d rats experienced significant constipation. For B, $n = 6$ retinas for all conditions. For C, NT $n = 12$ eyes, OHT $n = 9$ eyes, OHT EGCG 20 mg/kg/d $n = 7$ eyes; OHT EGCG 40 mg/kg/d $n = 12$ eyes, OHT GTP 80 mg/kg/d $n = 10$ eyes. * = $P < 0.05$, ** = $P < 0.01$, ***$P < 0.001$, NS = non-significant ($P > 0.05$); Student's t-test to control for A; One-way ANOVA with *Tukey's* HSD for B and C. For box plots, the center hinge represents the median with upper and lower hinges representing the first and third quartiles; whiskers represent 1.5 times the interquartile range.

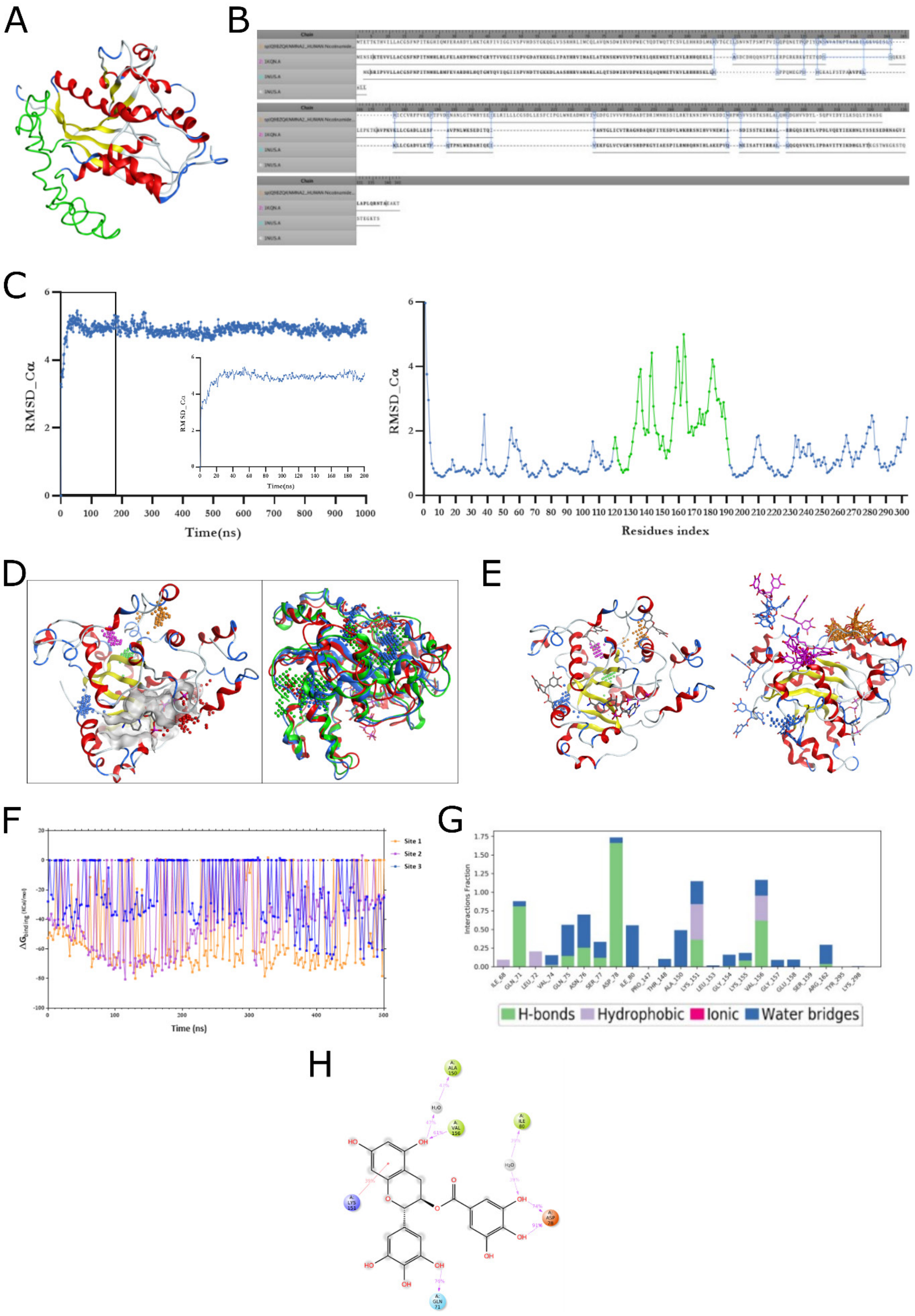
A
B
C
RMSD_Cα
Time(ns)
Residues index
D
E
F
Site 1
Site 2
Site 3
Time (ns)
G
Interactions Fraction
ILE_68
GLN_71
LEU_72
VAL_74
GLN_75
ASN_76
SER_77
ASP_78
ILE_80
PRO_147
THR_148
ALA_150
LYS_151
LEU_153
GLY_154
LYS_155
VAL_156
GLY_157
GLU_158
SER_159
ARG_162
TYR_295
LYS_298
H-bonds
Hydrophobic
Ionic
Water bridges
H

Fig. S4. In silico NMNAT2 homology model and EGCG binding

(**A**) Since NMNAT2 does not currently have a resolved crystal structure we generated a homology model using the crystal structures of NMNAT1 and NMNAT2 as a template as has been reported previously. The homology model was generated by I-Tasser. The generated protein model showed a low homology domain in the central region of the protein (green), in agreement with the literature (*9*) and the Alpha fold protein model. (**B**) Sequence alignment of NMNAT2 with NMNAT1 (1KQN) and NMNAT1(1NUS). (**C**) Molecular dynamic simulations demonstrated that the protein reached a stable conformation after 100 ns, converging around a fixed RMSD value (minimum c-alpha variation), with a higher fluctuation in the central region of the protein. (**D**) Using the SiteMap module of Schrödinger we identified different potential druggable binding-pocket, in addition to the catalytic pocket, on the NMANT2 surface. In 3 representative models the superposition of the structures demonstrated consensus between the identified pockets allowing for docking studies to validate the putative binding site of EGCG. (**E**) Docking results demonstrated that EGCG could be well accommodate in all pockets with ΔG values <-40 kcal/mol. A 500 ns molecular dynamic simulation was carried out to validate the binding mode and the protein-ligand complex stability. (**F**) The ΔG binding values demonstrated that only one ligand-protein complex (Site 1) could maintain a stable conformation during the entire MD simulation. EGCG molecules in binding sites 2 and 3 were more unstable, showing highly variable results in terms of occupation of the binding site, indicating that it is unlikely that EGCG can bind these two pockets (**G**) EGCG tended to optimize the pocket occupancies (Site 1) through several hydrophobic contacts and hydrogen bond interaction with the surrounding residues (*i.e.*, Gln71, Asp78, Lys151 and Val156), which were maintained for the entire simulation. (**H**) These are shown relative to the EGCG structure.

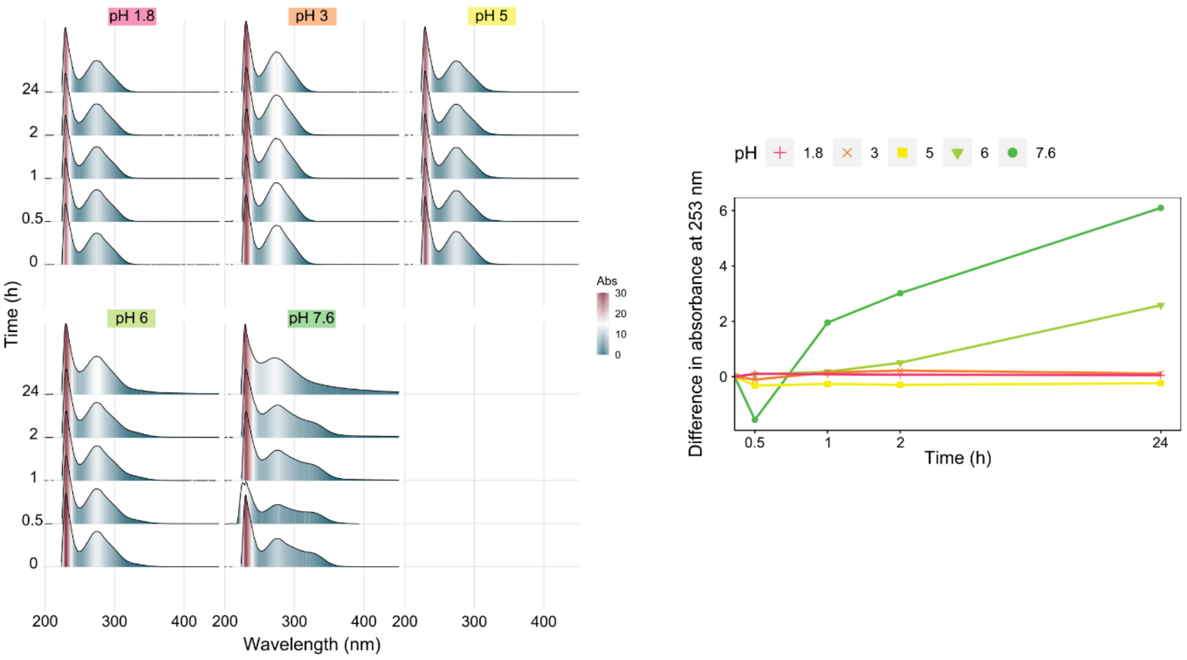

Fig. S5. EGCG stability with pH

When EGCG is dissolved in water or saline the solution browns over time indicating a possible breakdown of the EGCG molecule. **A)** We measured the absorbance profile of EGCG at 1 mM across a pH range relevant throughout the digestive system and **B)** display the change from time 0 at 253 nm. When dissolved in HBSS at an acidic pH, EGCG's absorbance profile remains stable over 24 h, but at more neutral pH the absorbance profile changes over 24 h. At pH 7.6, the absorbance profile at 0 h clearly differs in shape from those at the other pH ranges, indicating a rapid change to the EGCG molecule. The profile changes considerably over the 24 h time course with a high magnitude of change. This may underly the poor bioavailability of EGCG, since it will be rapidly broken down in the mouth when given as orally.

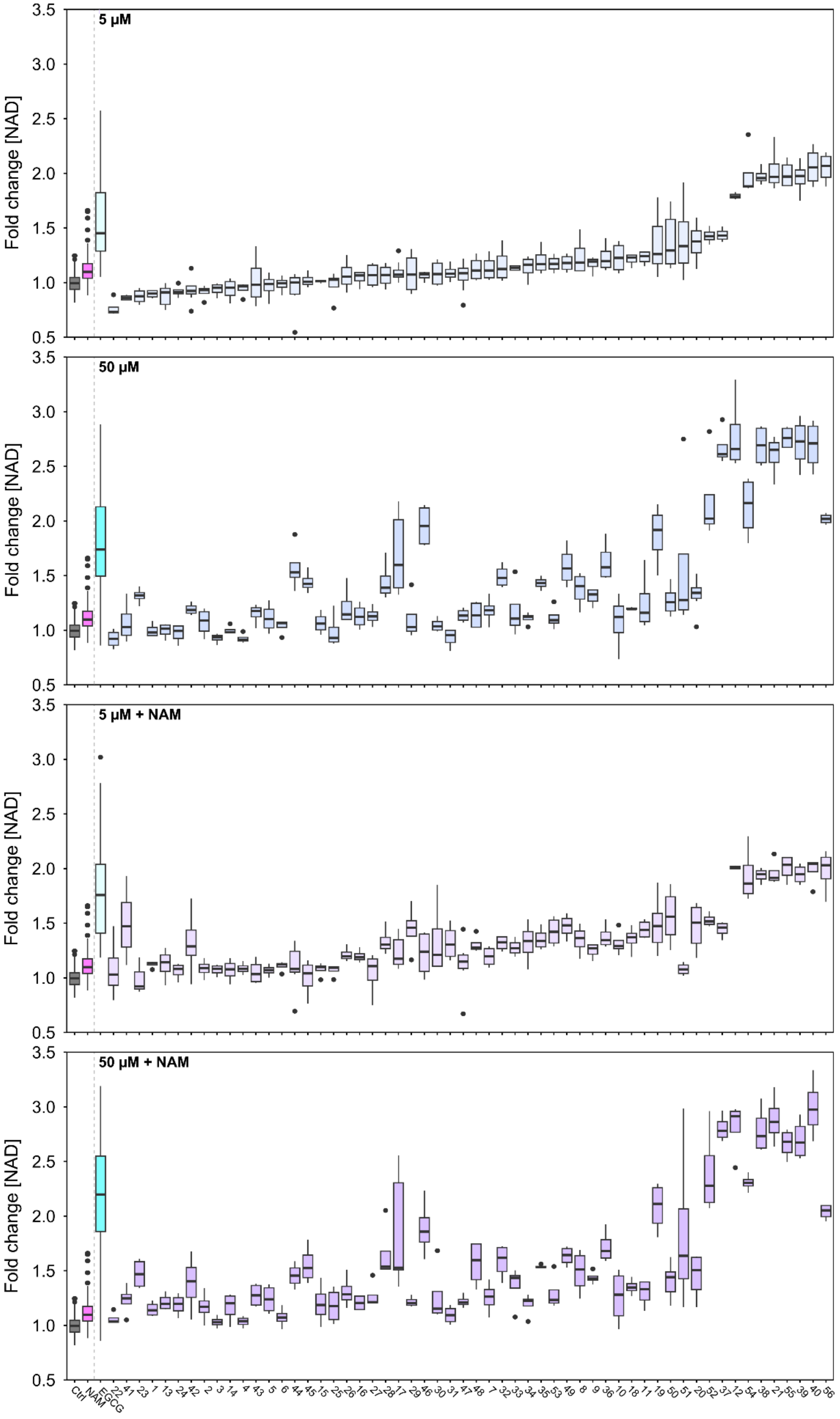
5 µM
50 µM
5 µM + NAM
50 µM + NAM
Fold change [NAD]
0.5
1.0
1.5
2.0
2.5
3.0
3.5
Ctrl NAM EGCG 22 41 23 1 13 24 42 2 3 14 4 43 5 6 44 45 15 25 26 16 27 28 17 29 46 30 31 47 48 7 32 33 34 35 53 49 8 9 36 10 18 11 19 50 51 20 52 37 12 54 38 21 55 39 40 56

Fig. S6. Compound NAD assays.

The capacity of EGCG analogues to boost NAD concentration was tested using a luminescence-based NAD assay in dissociated cortical neurons. NAD concentration relative to control was calculated for all analogues and compared to EGCG as a positive control. All compounds were tested at 5 μM and 50 μM, and in the presence of 100 μM nicotinamide, NAM to provide additional upstream substrate (which does not significantly increase NAD concentration alone). The results from all compounds are scaled to the untreated control (Ctrl) for their respective batch. Control, nicotinamide only (NAM), and EGCG only are displayed as the combined results of all batches within conditions. Compounds are ordered by mean fold change in NAD concentration in the 5 μM experiment condition. Statistical test results are displayed in **Supplementary Data S1**. For box plots, the center hinge represents the median with upper and lower hinges representing the first and third quartiles; whiskers represent 1.5 times the interquartile range.

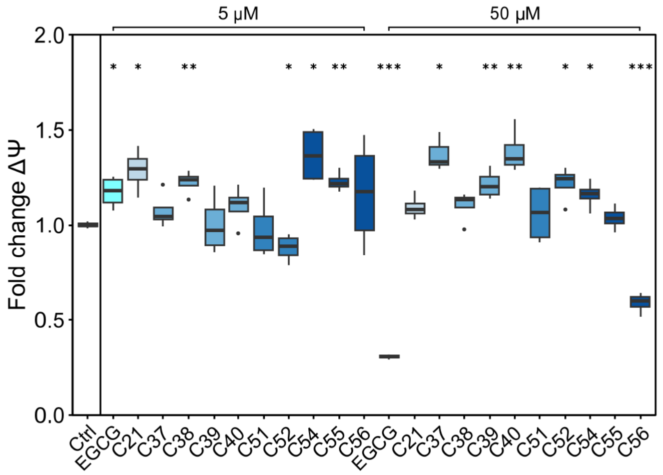

Fig. S7. Effects of top 10 compounds on mitochondrial membrane potential.
To investigate the effects of the top 10 compounds on mitochondria we incubated dissociated cortical cells with the compounds (or EGCG) at 5 and 50 µM for 2 hours, and labelled with JC-1 to measure the effect on mitochondrial membrane potential. EGCG and the compounds predominantly significantly increased membrane potential relative to controls at 5 and 50 µM. Compound 52 (at 5 µM), compound 56 (at 50 µM), and EGCG (at 50 µM) significantly lowered membrane potential relative to controls. Significance shown relative to controls. $n = 4$ for all conditions. * = $P < 0.05$, ** = $P < 0.01$, ***$P < 0.001$, NS = non-significant ($P > 0.05$); *Student's* t-test to control. For box plots, the center hinge represents the median with upper and lower hinges representing the first and third quartiles; whiskers represent 1.5 times the interquartile range.

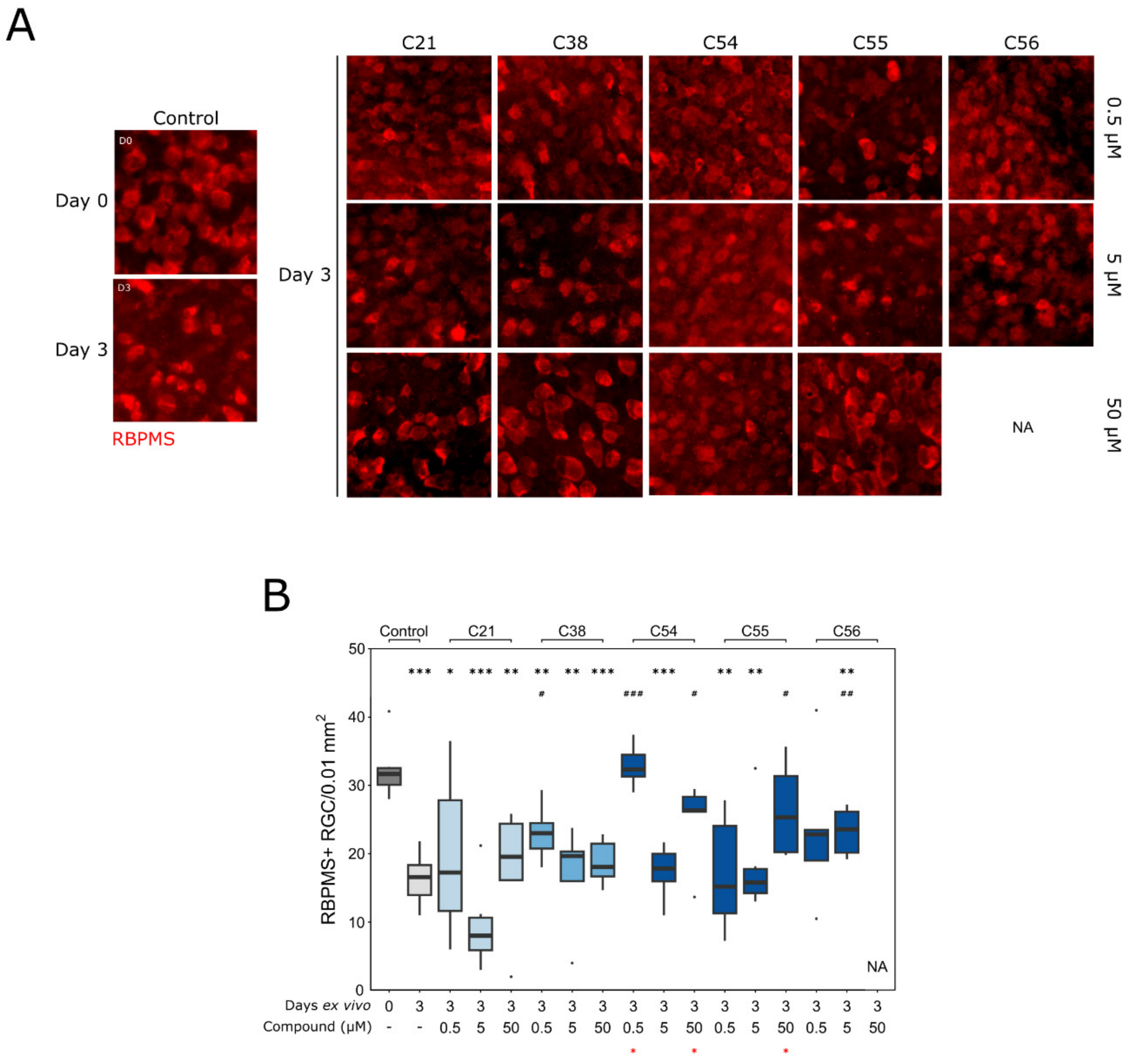

Fig. S8. Effects of top 10 compounds on RGC survival.

We tested the potential of these novel compounds to provide neuroprotection using a retinal explant model to quantify RGC survival following axotomy. Retinas were cultures for 3 days ex vivo with 5 selected compounds at 0.5, 5, and 50 µM. Example images of RGC survival (RBPMS labelling) are shown in (**A**) (retinas treated with **compound 56** at 50 µM could not be reliably imaged for quantification, denoted by NA). (**B**) Quantification of RGC density demonstrated that in untreated controls at 3 days ex vivo, significant RGC death occurred relative to naïve controls (0 days ex vivo). For the majority compound at 0.5 and 5 µM, RGC density was significantly reduced from 0 day controls and not significantly different from day 3 controls suggesting that at this concentration the majority of compounds had no effect on RGC survival. However, **compound 38** at 0.5 µM and **compound 56** at 5 µM were the exceptions, demonstrating a moderate/incomplete neuroprotection where RGC density was significantly reduced from day 0 but significantly increased from day 3. **Compound 54** at 0.5 µM and 0.5 µM, and **compound 55** at 50 µM demonstrated robust neuroprotection where RGC density was significantly higher than day 3 and was not significantly altered from day 0 (these are denoted by red * under the graph). Significance compared to day 0 control is denoted by *, significance compared to day 3 controls

is denoted by #. $*^{/\#} = P < 0.05$, $**^{/\#\#} = P < 0.01$, $***P < 0.001$; Student's t-test to control. For box plots, the center hinge represents the median with upper and lower hinges representing the first and third quartiles; whiskers represent 1.5 times the interquartile range.

Supplementary Table 1. Donor human retina details for explant model.

Donor ID	Age	Sex	Time from death to explant culture
1	77	M	48
2	77	F	48
3	43	M	24
4	72	M	60
5	42	M	30
6	21	M	40
7	54	M	48
8	70	F	48
9	64	M	37
10	49	F	50
