# Supplementary material for: NMNAT2 is a druggable target to drive neuronal NAD production
Source: Nat Commun. 2024 Jul 24;15:6256. doi: 10.1038/s41467-024-50354-5 (PMC11269627; doi:10.1038/s41467-024-50354-5)
Supplement: Supplementary file 3 — Description of Additional Supplementary Files [file 41467_2024_50354_MOESM3_ESM.pdf]

Description of Additional Supplementary Files

Supplementary Data 1. Data corresponding to Supplementary Figure 6 in table form including compound structures.

Supplementary Data 2. Data corresponding to statistical testing for Figure 8B.
