# Supplementary material for: NMNAT2 is a druggable target to drive neuronal NAD production
Source: Nat Commun. 2024 Jul 24;15:6256. doi: 10.1038/s41467-024-50354-5 (PMC11269627; doi:10.1038/s41467-024-50354-5)
Supplement: Supplementary file 6 — Reporting Summary [file 41467_2024_50354_MOESM6_ESM.pdf]

nature portfolio

Corresponding author(s): Pete Williams

Last updated by author(s): May 29, 2024

Reporting Summary

Nature Portfolio wishes to improve the reproducibility of the work that we publish. This form provides structure for consistency and transparency in reporting. For further information on Nature Portfolio policies, see our Editorial Policies and the Editorial Policy Checklist.

Statistics

For all statistical analyses, confirm that the following items are present in the figure legend, table legend, main text, or Methods section.

n/a	Confirmed	
☐	☒	The exact sample size (*n*) for each experimental group/condition, given as a discrete number and unit of measurement
☐	☒	A statement on whether measurements were taken from distinct samples or whether the same sample was measured repeatedly
☐	☒	The statistical test(s) used AND whether they are one- or two-sided<br>*Only common tests should be described solely by name; describe more complex techniques in the Methods section.*
☒	☐	A description of all covariates tested
☐	☒	A description of any assumptions or corrections, such as tests of normality and adjustment for multiple comparisons
☐	☒	A full description of the statistical parameters including central tendency (e.g. means) or other basic estimates (e.g. regression coefficient) AND variation (e.g. standard deviation) or associated estimates of uncertainty (e.g. confidence intervals)
☐	☒	For null hypothesis testing, the test statistic (e.g. *F*, *t*, *r*) with confidence intervals, effect sizes, degrees of freedom and *P* value noted<br>*Give P values as exact values whenever suitable.*
☒	☐	For Bayesian analysis, information on the choice of priors and Markov chain Monte Carlo settings
☒	☐	For hierarchical and complex designs, identification of the appropriate level for tests and full reporting of outcomes
☒	☐	Estimates of effect sizes (e.g. Cohen's *d*, Pearson's *r*), indicating how they were calculated

Our web collection on statistics for biologists contains articles on many of the points above.

Software and code

Policy information about availability of computer code

Data collection	no custom code central to manuscript
Data analysis	no custom code central to manuscript

For manuscripts utilizing custom algorithms or software that are central to the research but not yet described in published literature, software must be made available to editors and reviewers. We strongly encourage code deposition in a community repository (e.g. GitHub). See the Nature Portfolio guidelines for submitting code & software for further information.

Data

Policy information about availability of data

All manuscripts must include a data availability statement. This statement should provide the following information, where applicable:

- Accession codes, unique identifiers, or web links for publicly available datasets
- A description of any restrictions on data availability
- For clinical datasets or third party data, please ensure that the statement adheres to our policy

All data supporting the findings of this study are available within the paper and its Supplementary Information.

Research involving human participants, their data, or biological material

Policy information about studies with human participants or human data. See also policy information about sex, gender (identity/presentation), and sexual orientation and race, ethnicity and racism.

Reporting on sex and gender	Only sex is recorded for donor retina. Results are not sex disaggregated due to the low n number of samples
Reporting on race, ethnicity, or other socially relevant groupings	Donor retina do not have any accompanying information on race, ethnicity, or other socially relevant groupings
Population characteristics	NA
Recruitment	NA
Ethics oversight	Access to human eye tissues and patient samples is fully covered under "Studier av neuronal metabolism, biomarkörer och neuroprotektion vid glaukom" 2021-01036 (additions to base application 2020-01525; Pete Williams) approved by Regional Stockholm ethics committee.

Note that full information on the approval of the study protocol must also be provided in the manuscript.

Field-specific reporting

Please select the one below that is the best fit for your research. If you are not sure, read the appropriate sections before making your selection.

☒ Life sciences ☐ Behavioural & social sciences ☐ Ecological, evolutionary & environmental sciences

For a reference copy of the document with all sections, see nature.com/documents/nr-reporting-summary-flat.pdf

Life sciences study design

All studies must disclose on these points even when the disclosure is negative.

Sample size	Sample sizes for RGC density (cell counting) were chosen based on power calculations e.g. RGC survival (cell counting) (where Control = 33.14, disease = 20.63, SD = 7.05) sample size = 5 retina/group for Power = 0.80 and error rate = 0.05.
Data exclusions	Retinas which failed to achieve a significant IOP increase (>21 mmHg were excluded) as described for the model (DOI:10.1167/tvst.10.1.21). For the intravitreal rotenone model, injections which resulted in a lens injury or sub-retinal injection were excluded.
Replication	EGCG neuroprotection in explant retina was verified by two independent experimenters. EGCG induced NAD increase was replicated as a repeated control for analogue NAD testing.
Randomization	Animals were allocated to experimental groups prior to commencement of experiments
Blinding	For neurodegenerative measures, analysis was performed on animal/image IDs

Reporting for specific materials, systems and methods

We require information from authors about some types of materials, experimental systems and methods used in many studies. Here, indicate whether each material, system or method listed is relevant to your study. If you are not sure if a list item applies to your research, read the appropriate section before selecting a response.

Materials & experimental systems

n/a	Involved in the study	
☐	☒	Antibodies
☒	☐	Eukaryotic cell lines
☒	☐	Palaeontology and archaeology
☐	☒	Animals and other organisms
☒	☐	Clinical data
☒	☐	Dual use research of concern
☒	☐	Plants

Methods

n/a	Involved in the study	
☒	☐	ChIP-seq
☒	☐	Flow cytometry
☒	☐	MRI-based neuroimaging

Antibodies

Antibodies used	anti-βIII-tubulin: NovusBiological, cat #NB100-1612, lot # TUJ7947980<br>anti-RBPMS: NovusBiological, cat #NBP2-20112, lot # 42858

	anti-GFP: Abcam, cat # ab13970, lot #, GR3190550-8 and 1018753-4
Validation	anti-βIII-tubulin stains relevant layers in retina and has been validated in neuronal culture on the company website.<br>anti-RBPMS stains only retinal ganglion cell somas in the retina. We have validated this across species and against Thy1 reported mice. The antibody has been validated in numerous applications the company website<br>anti-GFP only gives positive staining in AAV injected tissue and we have also validated this in retina with CFP and YFP expression. The antibody has been validated in numerous applications the company website.

Animals and other research organisms

Policy information about studies involving animals; ARRIVE guidelines recommended for reporting animal research, and Sex and Gender in Research

Laboratory animals	C57BL/6J mice, used at age 11-18 weeks<br>Brown Norway rats (Rattus norvegicus), used at age 18-26 weeks<br>Nmnat2 gene-trap allele mouse lines, aged 13-22 months
Wild animals	NA
Reporting on sex	Sex was not considered in the study design
Field-collected samples	NA
Ethics oversight	Individual study protocols were approved by Stockholm's Committee for Ethical Animal Research (10389-2018 and 14053-2020) breeding an maintenance of Nmnat2 mice at The University of Cambridge (22-month old animals in this manuscript) was approved by home office license PPL P98A03BF9

Note that full information on the approval of the study protocol must also be provided in the manuscript.

Plants

Seed stocks	*Report on the source of all seed stocks or other plant material used. If applicable, state the seed stock centre and catalogue number. If plant specimens were collected from the field, describe the collection location, date and sampling procedures.*
Novel plant genotypes	*Describe the methods by which all novel plant genotypes were produced. This includes those generated by transgenic approaches, gene editing, chemical/radiation-based mutagenesis and hybridization. For transgenic lines, describe the transformation method, the number of independent lines analyzed and the generation upon which experiments were performed. For gene-edited lines, describe the editor used, the endogenous sequence targeted for editing, the targeting guide RNA sequence (if applicable) and how the editor was applied.*
Authentication	*Describe any authentication procedures for each seed stock used or novel genotype generated. Describe any experiments used to assess the effect of a mutation and, where applicable, how potential secondary effects (e.g. second site T-DNA insertions, mosiacism, off-target gene editing) were examined.*
